# Association Between Video-Based Telemedicine Visits and Medication Adherence Among Patients With Heart Failure: Retrospective Cross-Sectional Study

**DOI:** 10.2196/56763

**Published:** 2024-12-05

**Authors:** Yaguang Zheng, Samrachana Adhikari, Xiyue Li, Yunan Zhao, Amrita Mukhopadhyay, Carine E Hamo, Tyrel Stokes, Saul Blecker

**Affiliations:** 1 New York University Rory Meyers College of Nursing, 433 1st Avenue, New York, NY, 10010, United States, 1 212-998-5170; 2 Department of Population Health, New York University Grossman School of Medicine, New York, NY, United States; 3 Leon H. Charney Division of Cardiology, Department of Medicine, New York University Grossman School of Medicine, New York, NY, United States

**Keywords:** telemedicine, medication adherence, heart failure, systolic dysfunction, medical therapy, telehealth, remote monitoring, self-management

## Abstract

**Background:**

Despite the exponential growth in telemedicine visits in clinical practice due to the COVID-19 pandemic, it remains unknown if telemedicine visits achieved similar adherence to prescribed medications as in-person office visits for patients with heart failure.

**Objective:**

Our study examined the association between telemedicine visits (vs in-person visits) and medication adherence in patients with heart failure.

**Methods:**

This was a retrospective cross-sectional study of adult patients with a diagnosis of heart failure or an ejection fraction of ≤40% using data between April 1 and October 1, 2020. This period was used because New York University approved telemedicine visits for both established and new patients by April 1, 2020. The time zero window was between April 1 and October 1, 2020, then each identified patient was monitored for up to 180 days. Medication adherence was measured by the mean proportion of days covered (PDC) within 180 days, and categorized as adherent if the PDC was ≥0.8. Patients were included in the telemedicine exposure group or in-person group if all encounters were video visits or in-person office visits, respectively. Poisson regression and logistic regression models were used for the analyses.

**Results:**

A total of 9521 individuals were included in this analysis (telemedicine visits only: n=830 in-person office visits only: n=8691). Overall, the mean age was 76.7 (SD 12.4) years. Most of the patients were White (n=6996, 73.5%), followed by Black (n=1060, 11.1%) and Asian (n=290, 3%). Over half of the patients were male (n=5383, 56.5%) and over half were married or living with partners (n=4914, 51.6%). Most patients’ health insurance was covered by Medicare (n=7163, 75.2%), followed by commercial insurance (n=1687, 17.7%) and Medicaid (n=639, 6.7%). Overall, the average PDC was 0.81 (SD 0.286) and 71.3% (6793/9521) of patients had a PDC≥0.8. There was no significant difference in mean PDC between the telemedicine and in-person office groups (mean 0.794, SD 0.294 vs mean 0.812, SD 0.285) with a rate ratio of 0.99 (95% CI 0.96-1.02; *P*=.09). Similarly, there was no significant difference in adherence rates between the telemedicine and in-person office groups (573/830, 69% vs 6220/8691, 71.6%), with an odds ratio of 0.94 (95% CI 0.81-1.11; *P*=.12). The conclusion remained the same after adjusting for covariates (eg, age, sex, race, marriage, language, and insurance).

**Conclusions:**

We found similar rates of medication adherence among patients with heart failure who were being seen via telemedicine or in-person visits. Our findings are important for clinical practice because we provide real-world evidence that telemedicine can be an approach for outpatient visits for patients with heart failure. As telemedicine is more convenient and avoids transportation issues, it may be an alternative way to maintain the same medication adherence as in-person visits for patients with heart failure.

## Introduction

Approximately 6.7 million American adults experience heart failure [1], which is a leading cause of morbidity and mortality globally [2]. There are currently four classes of guideline-directed medical therapies (GDMTs) shown to improve outcomes for patients with heart failure, which include β-blockers (BBs), angiotensin-converting enzyme inhibitors/angiotensin receptor blockers (ACEI/ARBs) or angiotensin receptor neprilysin inhibitors (ARNIs), mineralocorticoid receptor antagonists (MRAs), and sodium glucose cotransporter 2 inhibitors (SGLT2Is) [3]. Adherence to these prescribed therapies has been associated with reduced cost, reduced heart failure–related morbidity and mortality, and improved quality of life for patients with heart failure [4-7].

Patients with heart failure have increasingly been using remote care as part of their treatment course [8]. A systemic review of randomized clinical trials indicated that the use of telemedicine in the management of heart failure appeared to lead to similar health outcomes as face-to-face delivery of care [9]. However, the majority of these studies were conducted in randomized controlled trials (RCTs). With the exponential growth in telemedicine visits for outpatient care, few studies have reported real-world evidence (eg, using data from electronic medical records) on the association between telemedicine visits and health outcomes among outpatients. Studies using electronic medical record data have shown that telemedicine has improved medication adherence among patients seen in an outpatient gastroenterology clinic [10], as well as an improvement in mean monthly tobacco treatment for inpatient counseling and an increase in outreach visits in the telehealth period compared with the pretelehealth period [11]. One study found that hospitalized patients with heart failure who received an outpatient follow-up either via telemedicine or in-person had a lower 30-day readmission rate than those who received no follow-up [12]. Telehealth has reduced wait times for appointments and may increase clinician visit frequency, which may help improve medication adherence [13]. However, to our knowledge, no study has examined the potential impact of the type of visits on medicine adherence among patients with heart failure using electronic medical records. The difference between an RCT and the study using real-world data with respect to adherence is that adherence in an RCT is enforced to ensure any lack of efficacy of the tested drug is not due to low adherence [14,15]. Therefore, our study aimed to examine the association between telemedicine visits versus in-person visits on medication adherence to heart failure GDMT.

## Methods

### Study Design

This was a retrospective, cross-sectional study of adult patients with heart failure or an ejection fraction of ≤40% using the electronic health record data from New York University Langone Health (NYULH) system [16], a large academic health care system with a telehealth infrastructure in New York City. The NYULH system includes 235 facilities in New York City’s 5 boroughs, Long Island, New Jersey, Westchester County, Putnam County, and Duchess County. The participating sites include academic practices, community-based practices, and federally qualified health centers, serving an ethnically and socially diverse population. The data were retrieved from patients who had at least one outpatient encounter with a cardiologist, internist, subspeciality provider, or primary care provider between April 1 and October 1, 2020. This period of time was used because New York University approved telemedicine visits for both established and new patients by April 1, 2020. The time zero window was between April 1 and October 1, 2020, and then each identified patient was monitored for up to 180 days.

### Ethical Considerations

The study was approved by the institutional review board at NYULH (i19-00131). Informed consent was not applicable, as this was a secondary data analysis. Study data were deidentified and compensation type and amount for human subjects research were not applicable.

### Inclusion and Exclusion Criteria

Patients were included if (1) they had a diagnosis of heart failure or an ejection fraction of ≤40% based on a transthoracic echocardiogram [17] and (2) they were prescribed any or all the following GDMT categories: BBs, ACEI/ARBs, ARNIs, MRAs, and SGLT2Is. Patients were excluded if (1) they had mixed telemedicine and in-person office visits or (2) their medications’ overall prescribing duration was <28 days, because our interest was in characterizing adherence to chronic GDMT regimens.

### Measures

#### Primary Outcome: Medication Adherence

The primary outcome was adherence to the GDMT, measured by the proportion of days covered (PDC), which is a ratio between the number of days a medication is dispensed for a patient divided by the number of days it is prescribed. The PDC was measured for a period of 180 days. Early terminated prescriptions of less than 28 days were excluded. The PDC was calculated for each GDMT, and the average PDC across GDMT categories was assessed as a continuous outcome, and standardized to the number of days covered over a total of 180 days. We also evaluated the PDC as a binary outcome where a PDC≥0.8 was defined as adherent, which is commonly used as the cutoff for medication adherence [18-20].

#### Primary Exposure Measure: Types of Visits (Telemedicine vs In-Person Office)

Patients who had outpatient encounters at NYULH between April 1 and October 1, 2020, were divided into two groups. Patients who only had telemedicine visits during this period were in the telemedicine group, while patients who only had in-person visits were in the in-person visit group. Telemedicine visits were defined as ambulatory care video encounters with a cardiologist, internist, subspecialty provider, or primary care provider. The purely telephone visit encounters were not counted as telemedicine visits because telemedicine at NYULH is exclusively video-based [21]. In-person office visits were defined as office visit encounters with a cardiologist, internist, subspecialty provider, or primary care provider.

#### Covariates

There were 4 types of covariates. First, demographic covariates included age, sex (male or female), race (White, Black, Asian, Pacific Islander/Native Hawaiian/American Indian, or other), marital status (married/living with partners, or single/separated/other), preferred language (English, Spanish, Russian, or other), and insurance status (Medicare, Medicaid, commercial, or other) [22]. Second, health care usage measures included the number of hospitalizations or outpatient visits defined as visit encounters with a cardiologist, internist, subspecialty providers in cardiology, or primary care provider in the past year. The third covariate was the Elixhauser comorbidity score, a method categorizing comorbidities of patients based on the *International Classification of Diseases*’ (*ICD*) health code of comorbidities (eg, hypertension, cardiac arrhythmias, obesity, valvular disease, peripheral vascular disorders, diabetes, chronic pulmonary disease, and chronic kidney disease) [23]. We used the standard *ICD-10-CM* (*International Classification of Diseases, Tenth Revision, Clinical Modification*) for each comorbidity (eg, hypertension such as the *IDC-10-CM* codes 401.1, 401.9, I10.x, I11.x-I13.x, and I15.x; chronic kidney disease such as 403.11, I12.0, I13.1). Each comorbidity category was dichotomous and reported as either present or not [23]. Fourth, the Agency for Healthcare Research and Quality neighborhood social economic status (SES) index was computed based on the American Community Survey variables, which combined information on crowding, property value, unemployment, poverty level, income, and education [22].

### Statistical Analysis

All statistical analyses were conducted in R version 4.2.2 (R Foundation for Statistical Computing). A histogram plot was used to assess the distribution of the continuous PDC. Since the PDC can have negative values, we used a robust Poisson regression to examine the association between types of visits (telemedicine vs in-person office) and the average PDC. Based on the distribution of the outcome, the sandwich estimator was used to obtain the robust SE and *P* values. The rate ratio and 95% CI were calculated and reported. Logistic regression was used to examine the association between types of visits (telemedicine vs in-person office) and adherence to GDMTs as a binary outcome (PDC≥0.8). The odds ratio (95% CI) was calculated and reported. Both Poisson and logistic regression models included covariates in a stepped fashion as follows: model 1 was unadjusted for covariates; model 2 adjusted for sociodemographic characteristics, including age, sex, race, marriage, language, and insurance; model 3 incorporated the comorbidity index; model 4 further added the health care visits, including the number of hospitalizations, number of outpatient visits, and number of primary care provider visits in the past year; and model 5 further added the neighborhood SES index.

## Results

**Figure 1.**
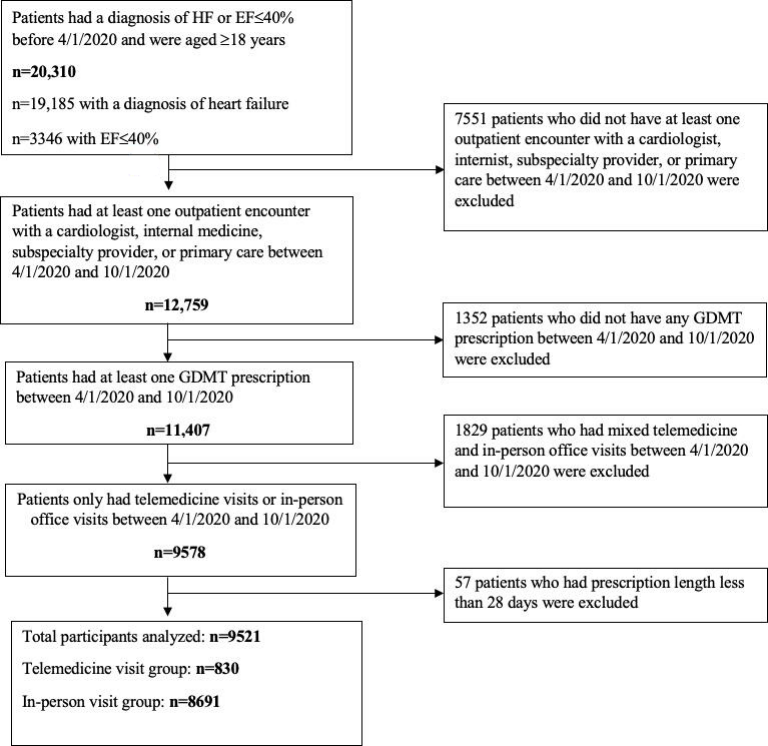
Flowchart of the study design. EF: ejection fraction; GDMT: guideline-directed medical therapy; HF: heart failure.

A total of 9521 individuals with heart failure were included in this analysis, with 830 individuals in the telemedicine visits group and 8691 individuals in the in-person office visits group (Figure 1, Table 1). Overall, the mean age was 76.7 (SD 12.4) years. Most of the patients were White (n=6996, 73.5%), followed by Black (n=1060, 11.1%) or Asian (n=290, 3%). Over half of the patients were male (n=5383, 56.5%), and over half were married or living with partners (n=4914, 51.6%). Most patients’ health insurance was covered by Medicare (n=7163, 75.2%), followed by commercial insurance (n=1687, 17.7%) and Medicaid (n=639, 6.7%). Most of the patients had comorbid medical conditions including hypertension (n=7892, 82.9%), cardiac arrhythmias (n=5691, 59.8%), obesity (n=3473, 36.5%), valvular disease (n=3374, 35.4%), peripheral vascular disorders (n=3205, 33.7%), diabetes without complications (n=2824, 29.7%), diabetes with complications (n=2017, 21.2%), chronic pulmonary disease (n=2360, 24.8%), and chronic kidney disease (n=1953, 20.5%). The rate of prescription for each GDMT category included was as follows: BB (n=7803, 82%), ACEI/ARB (n=6167, 64.8%), ARNI (n=1421, 14.9%), MRA (n=2017, 21.2%), and SGLT2I (n=667, 7%).

**Table 1.** Sample characters by types of visits.

Baseline characteristics	Overall (n=9521)	In-person visit (n=8691)	Telemedicine visit (n=830)	*P* value
Age (years), mean (SD)	76.7 (12.4)	77.1 (12.2)	72.6 (14.3)	<.001
Sex, n (%)				.87
Female	4138 (43.5)	3780 (43.5)	358 (43.1)	
Male	5383 (56.5)	4911 (56.5)	472 (56.9)	
Race, n (%)				.04
White	6996 (73.5)	6423 (73.9)	573 (69)	
African American (Black)	1060 (11.1)	950 (10.9)	110 (13.3)	
Asian	290 (3)	263 (3)	27 (3.3)	
Other race	725 (7.6)	646 (7.4)	79 (9.5)	
Pacific Islander/Native Hawaiin/American Indian	28 (0.3)	27 (0.3)	1 (0.1)	
Refused/unknown	422 (4.4)	382 (4.4)	40 (4.8)	
Language, n (%)				<.001
English	7835 (82.3)	7088 (81.6)	747 (90)	
Spanish	442 (4.6)	415 (4.8)	27 (3.3)	
Russian	733 (7.7)	702 (8.1)	31 (3.7)	
Other	491 (5.2)	466 (5.4)	25 (3)	
Marital status, n (%)				.005
Married/living with partners	4914 (51.6)	4441 (51.1)	473 (57)	
Single/separated/other	4407 (46.3)	4066 (46.8)	341 (41.1)	
Unknown	200 (2.1)	184 (2.1)	16 (1.9)	
Insurance, n (%)				<.001
Medicare	7163 (75.2)	6648 (76)	515 (62)	
Medicaid	639 (6.7)	576 (6.6)	63 (7.6)	
Commercial	1687 (17.7)	1438 (16.5)	249 (30)	
Other	11 (0.1)	11 (0.1)	0 (0)	
Health care visits in the past year, mean (SD)				
Number of hospitalizations	0.222 (0.669)	0.211 (0.641)	0.334 (0.909)	<.001
Number of outpatient visits	3.98 (3.35)	4.01 (3.39)	3.67 (2.94)	.002
Number of primary care provider visits	0.0118 (0.258)	0.0120 (0.262)	0.00964 (0.202)	.76
Neighborhood SES^a^ index, mean (SD)	55.9 (4.51)	55.8 (4.44)	56.5 (5.09)	<.001
Comorbid conditions				
Comorbidity^b^ index (Elixhauser), mean (SD)	12.6 (7.14)	12.6 (7.06)	13.0 (7.93)	.17
Congestive heart failure, n (%)	8723 (91.6)	7958 (91.6)	765 (92.2)	.59
Ejection fraction, mean (SD)	49.3 (14.5)	49.2 (14.5)	49.9 (15.3)	.36
Hypertension, uncomplicated; n (%)	7892 (82.9)	7266 (83.6)	626 (75.4)	<.001
Cardiac arrhythmias, n (%)	5691 (59.8)	5211 (60)	480 (57.8)	.25
Obesity, n (%)	3473 (36.5)	3201 (36.8)	272 (32.8)	.02
Valvular disease, n (%)	3374 (35.4)	3115 (35.8)	259 (31.2)	.008
Peripheral vascular disorders, n (%)	3205 (33.7)	2956 (34)	249 (30)	.02
Diabetes (uncomplicated), n (%)	2824 (29.7)	2594 (29.8)	230 (27.7)	.21
Diabetes (complicated), n (%)	2017 (21.2)	1851 (21.3)	166 (20)	.41
Chronic pulmonary disease, n (%)	2360 (24.8)	2171 (25)	189 (22.8)	.17
Chronic kidney disease, n (%)	1953 (20.5)	1779 (20.5)	174 (21)	.77
Prescribed GDMT^c^				
Prescribed ACEI/ARB^d^, n (%)	6167 (64.8)	5675 (65.3)	492 (59.3)	<.001
Prescribed ARNI^e^, n (%)	1421 (14.9)	1292 (14.9)	129 (15.5)	.64
Prescribed MRA^f^, n (%)	2017 (21.2)	1811 (20.8)	206 (24.8)	.008
Prescribed BB^g^, n (%)	7803 (82)	7120 (81.9)	683 (82.3)	.83
Prescribed SGLT2I^h^, n (%)	667 (7)	600 (6.9)	67 (8.1)	.23

a SES: social economic status score.

b Comorbidity index was calculated based on *International Statistical Classification of Diseases, Tenth Revision* (*ICD-10*) codes from encounter, hospitalization, and problem data before and on baseline.

c GDMT: guideline-directed medical therapy.

d ACEI/ARB: angiotensin-converting enzyme inhibitor/angiotensin receptor blocker.

e ARNI: angiotensin receptor neprilysin inhibitor.

f MRA: mineralocorticoid receptor antagonist.

g BB: β-blocker.

h SGLT2: sodium glucose cotransporter 2 inhibitor.

Baseline characteristics of patients in telemedicine and in-person office visit groups are displayed in Table 1. Individuals in the telemedicine visits group were younger (72.6 vs 77.1 years; *P*<.001), with a higher proportion of people who were African American (110/830, 13.3% vs 950/8691, 10.9%) or Asian (27/830, 3.3% vs 263/8691, 3.0%; *P*=.04), preferred speaking English (747/830, 90% vs 7088/8691, 81.6%; *P*<.001), and were married or living with partners (473/830, 57% vs 4441/8691, 51.1%; *P*=.005) compared to those in the in-person visits group. However, a lower proportion of patients in the telemedicine group had Medicare insurance (515/830, 62% vs 6648/8691, 76.5%; *P*=.005) or were prescribed ACEI/ARB therapy (492/830, 59.3% vs 5675/8691, 65.3%; *P*<.001). Individuals in the telemedicine visit group had a higher number of hospitalizations in the past year (mean 0.334, SD 0.909 vs mean 0.211, SD 0.641; *P*<.001) and a lower number of outpatient visits in the past year (mean 3.67, SD 2.94 vs mean 4.01, SD 3.39; *P*<.001).

**Figure 2.**
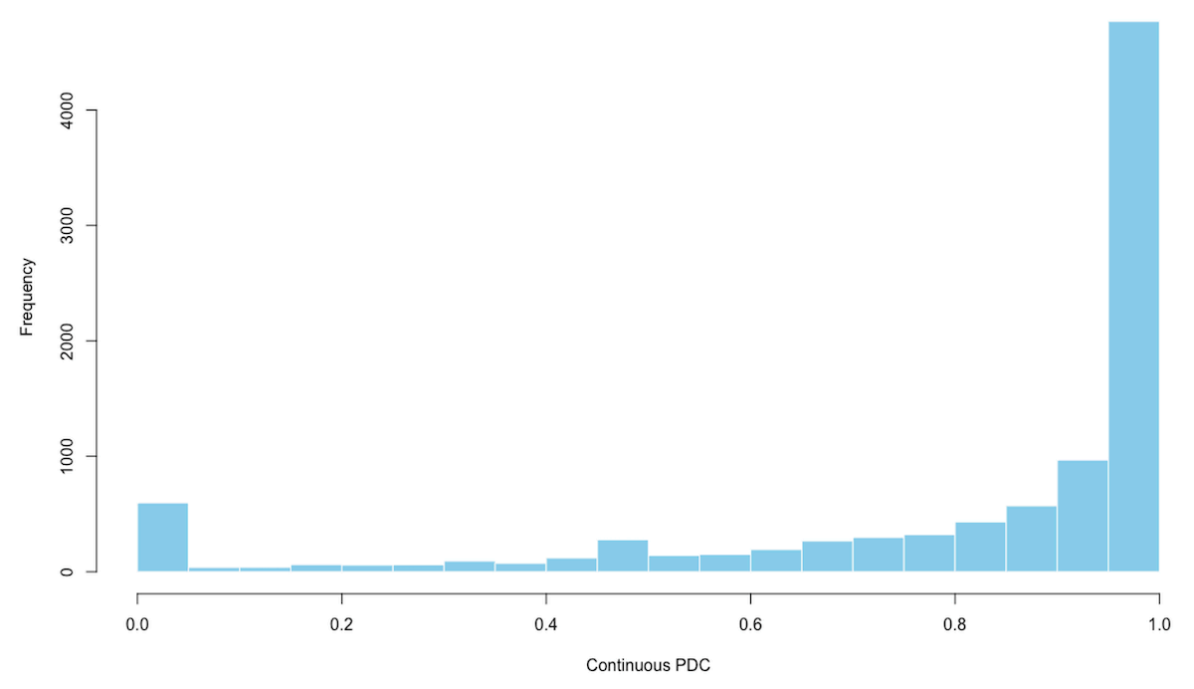
Distribution of the continuous PDC showing medication adherence for patients. PDC: proportion of days covered.

A histogram plot was used to assess the distribution of the continuous PDC (Figure 2). Overall, the average PDC was 0.81 (SD 0.286) and 71.3% (6793/9521) of patients had a PDC≥0.8 (Table 2). In the unadjusted model, the PDC between telemedicine visits and in-person office visits groups was not statistically different (mean 0.794, SD 0.294 vs mean 0.812, SD 0.285), with a rate ratio of 0.98 (95% CI 0.95-1.00; *P*=.09) (Table 3). The ratio of the PDC by types of visits remained similar after adjusting for demographic covariates including age, sex, race, marriage, language, and insurance (rate ratio 0.99, 95% CI 0.96-1.01; *P*=.34); demographics and comorbidity index (rate ratio 0.99, 95% CI 0.96-1.01; *P*=.34); demographics, comorbidity index, and health care usage in the past year, including the number of hospitalizations, number of outpatient visits, and number of primary care provider visits (rate ratio 0.99, 95% CI 0.96-1.02; *P*=.44); and demographics, comorbidity index, health care usage in the past year, and neighborhood SES index (rate ratio 0.99, 95% CI 0.97-1.02; *P*=.49).

**Table 2.** Medication adherence by types of visits.

Overall (n=9521)	In-person visit (n=8691)	Telemedicine visit (n=830)	*P* value
PDC^a^				.08
Mean (SD)	0.810 (0.286)	0.812 (0.285)	0.794 (0.294)	
Median (IQR)	0.960 (0.74-1.00)	0.960 (0.75-1.00)	0.940 (0.68-1.00)
Adherent (PDC≥0.8), n (%)				.13
Yes	6793 (71.3)	6220 (71.6)	573 (69)	
No	2728 (28.7)	2471 (28.4)	257 (31)

a PDC: proportion of days covered.

**Table 3.** The association between telemedicine visits and medication adherence for patients with heart failure.

Model	Continuous PDC^a^ outcome		Binary PDC outcome	
	Rate ratio (95% CI)	*P* value	Odds ratio (95% CI)	*P* value
Model 1, unadjusted	0.98 (0.95-1.00)	.09	0.89 (0.76-1.03)	.12
Model 2, adjusting for demographics	0.99 (0.96-1.01)	.34	0.93 (0.80-1.10)	.40
Model 3, adjusting for demographics and comorbidity index	0.99 (0.96-1.01)	.34	0.93 (0.80-1.09)	.39
Model 4, adjusting for demographics, comorbidity index, and health care visits in the past year	0.99 (0.96-1.02)	.44	0.94 (0.81-1.11)	.48
Model 5, adjusting for demographics, comorbidity index, health care visits in the past year, and neighborhood SES^b^ index	0.99 (0.97-1.02)	.49	0.96 (0.82-1.13)	.65

a PDC: proportion of days covered.

b SES: social economic status score.

Similarly, without adjusting covariates, there was no significant difference in the percent of PDC ≥0.8 between the telemedicine visits and in-person office visits groups (573/830, 69% vs 6220/8691, 71.6%), with the odds ratio for adherence of 0.89 (95% CI 0.76-1.03; *P*=.12). The odds ratio of medication adherence to GDMT by types of visits remained the same after adjusting for demographics (odds ratio 0.93, 95% CI 0.80-1.10; *P*=.40); demographics and comorbidity index (odds ratio 0.93, 95% CI 0.80-1.09; *P*=.39); demographics, comorbidity index, and health care usage in the past year (odds ratio 0.94, 95% CI 0.81-1.11; *P*=.48); and demographics, comorbidity index, health care usage in the past year, and neighborhood SES index (odds ratio 0.96, 95% CI 0.82-1.13; *P*=.65).

## Discussion

Using the electronic medical record data from a large academic health care system, our results indicate that patients with heart failure had similar medication adherence to GDMT between telemedicine and in-person office visits. Our findings are important because we provide real-world evidence that, for patients with heart failure, telemedicine can be an approach to outpatient visits, which may be an alternative way to maintain the same medication adherence as in-person visits. The randomized clinical trials summarized by a systematic review conducted prior to COVID-19 indicated that the use of telemedicine in the management of heart failure appears to lead to similar health outcomes as face-to-face or telephone delivery of care [9]. Our study, using real-world data from electronic medical records, shows that patients with heart failure have no differences in medication adherence between telemedicine and in-person office visits. Despite the exponential growth in telemedicine visits for outpatient care, limited studies have examined the effect of telemedicine visits on medication adherence among patients with heart failure using electronic medical records. One study found that hospitalized patients with heart failure, who received outpatient follow-up via telemedicine, had a lower 30-day readmission rate than those who received no follow-up [12]; however, this study did not examine medication adherence. For a different medical condition, one study using electronic medical record data indicated that telemedicine improved medication adherence among patients seen in an outpatient gastroenterology clinic [10].

Our results indicate that, compared with participants who had gone to in-person office visits, the participants in the telemedicine group were younger, more likely to be African American or Asian, preferred speaking English, were married or living with partners, and had lower rates of Medicare insurance. The data from the NYULH system are uniquely suited to explore the digital disparities in telemedicine, given its well-developed digital health infrastructure [24]. Previous studies reported that the proportion of young African American individuals accessing care through telemedicine increased after COVID-19 [24,25]. Our finding that more individuals who had only telemedicine visits preferred speaking English is consistent with a prior finding [25], which might be due to the fact that patient portals are only developed in English [26]. Similarly, our finding that a higher proportion of patients in the telemedicine group were married or living with a partner, compared to the in-person visit group, is consistent with prior findings [27]. Our study adds to the literature, showing that patients with heart failure who were younger adults, African American or Asian American, preferred speaking English, or were married or living with partners may benefit from telemedicine visits for medication adherence to GDMT.

The limitations of this study include unavailable variables related to digital literacy in the electronic health record dataset and the homogeneity of the patient population that was mostly White and had health insurance covered by Medicare*,* limiting generalization to other populations such as individuals with Medicaid or differing digital health literacy. Additionally, medication adherence was defined based on pharmacy fill data, which might not accurately reflect true medication adherence to GDMT, though the PDC is a commonly used measure for medication adherence [18-20]. Some people may not meet the criteria for GDMT, but adherence is still important if therapy is prescribed. Moreover, causal inference cannot be made due to the cross-sectional nature of this study. Furthermore, during most periods of the study, SGLT2Is had not been approved for heart failure; we also included patients, including those with heart failure with preserved ejection fraction, for whom some of these medications may not be part of GDMT. However, our study focuses on adherence to medications prescribed by a provider, and we presume adherence to prescribed medications is important regardless of the indications for prescribing. We admit that the way the primary outcome adherence is measured does not take into account the dosage or reaching targets for GDMT, and someone on the lowest dose of all therapies would get a perfect score. However, the strength of the study is that we provide real-world evidence for the application of telemedicine in clinical practice. Future research should examine telemedicine effects gathered from multiple health systems.

In summary, using the electronic medical record data from a large academic health care system, our study indicates that patients with heart failure have no differences in medication adherence between telemedicine and in-person office visits. Our study also indicates that patients who were younger, were African American or Asian, preferred speaking English, or were married or living with partners might particularly benefit from telemedicine visits. Our findings are important for clinical practice because we provide real-world evidence that, for patients with heart failure, telemedicine visits can be an approach for outpatient visits. As telemedicine is more convenient and avoids transportation issues, it may be an alternative way to maintain the same medication adherence to GDMT as in-person visits for patients with heart failure.

## Abbreviations

ACEI: angiotensin-converting enzyme inhibitor
ARB: angiotensin receptor blocker
ARNI: angiotensin receptor neprilysin inhibitor
BB: β-blocker
GDMT: guideline-directed medical therapy
*ICD*: *International Classification of Diseases*
*ICD-10-CM*: *International Classification of Diseases, Tenth Revision, Clinical Modification*
MRA: mineralocorticoid receptor antagonist
NYULH: New York University Langone Health
PDC: proportion of days covered
RCT: randomized controlled trial
SES: social economic status
SGLT2I: sodium glucose cotransporter 2 inhibitor
